# Changes in the Incidence and Human Papillomavirus-Positive Portion of Oropharyngeal Squamous Cell Carcinoma in Hong Kong

**DOI:** 10.3390/cancers16010226

**Published:** 2024-01-03

**Authors:** Zigui Chen, Amy B. W. Chan, Lok-Sang Kam, Man-Hin Chan, Jason Y. K. Chan, Wai-Tung Lee, Chit Chow, Siaw S. Boon, Chichao Xia, Brian Lam, Suki Lam, Rita W. Y. Ng, Wendy C. S. Ho, Eddy W. H. Lam, Christopher K. C. Lai, Paul K. S. Chan

**Affiliations:** 1 Department of Microbiology, The Chinese University of Hong Kong, Hong Kong SAR, China; zigui.chen@cuhk.edu.hk (Z.C.); boonss@cuhk.edu.hk (S.S.B.); chichaoxia@cuhk.edu.hk (C.X.); ritang@cuhk.edu.hk (R.W.Y.N.); cc_wendy@cuhk.edu.hk (W.C.S.H.); chris.kclai@cuhk.edu.hk (C.K.C.L.); 2 Department of Anatomical and Cellular Pathology, The Chinese University of Hong Kong, Hong Kong SAR, China; abwchan@cuhk.edu.hk (A.B.W.C.); chit@cuhk.edu.hk (C.C.); 3 Department of Anatomical Pathology, Tuen Mun Hospital, Hong Kong SAR, China; 4 Department of ENT, Yan Chai Hospital, Hong Kong SAR, China; cmh888@ych.ha.org.hk (M.-H.C.); lmw845@ha.org.hk (B.L.); enthndreddylam@gmail.com (E.W.H.L.); 5 Department of Otorhinolaryngology, Head and Neck Surgery, The Chinese University of Hong Kong, Hong Kong SAR, China; 6 Department of Pathology, Queen Elizabeth Hospital, Hong Kong SAR, China; lwt676@ha.org.hk

**Keywords:** HPV, oropharyngeal cancer, tonsil cancer, head and neck cancers, OPSCC, Hong Kong, Chinese

## Abstract

**Simple Summary:**

Oral infection with high-risk human papillomaviruses is one of the known risk factors for oropharyngeal cancer. Human papillomavirus-related oropharyngeal cancer is a rising trend in many Western countries. Hong Kong is a vibrant Chinese cosmopolitan city in East Asia where data on the trend of change in human papillomavirus-related oropharyngeal cancer are not available. This study found that oropharyngeal cancer cases have increased persistently over the last three decades in Hong Kong, despite a notable decrease in other head and neck cancers such as the laryngeal cancer. By testing a series of cancer samples collected over the past several years, this study found that the proportion of oropharyngeal cancer infected with high-risk human papillomaviruses has increased substantially over the last decade. Strategies to prevent oral human papillomavirus infection and its associated diseases including oropharyngeal cancer are urgently needed. Research on the early detection of oropharyngeal cancer is a priority.

**Abstract:**

The incidence of human papillomavirus (HPV)-associated oropharyngeal squamous cell carcinoma (OPSCC) is rising in the West, but little is known in Asia. This study elucidated changes in the incidence and HPV-positive portion of OPSCC in Hong Kong. Data from population-based cancer registry were used to analyze the incidence of OPSCC in association with other head and neck cancers. Archived tumor tissues were tested for HPV. From 1986 to 2020, there was a marked decrease in the incidence of nasopharyngeal and laryngeal cancers, but a persistent increase in OPSCC from 36 cases in 1986 to 116 cases in 2020. The average positive rate for high-risk HPV was 36.1% (112/310) among OPSCC diagnosed in 2010–2020. The HPV-positive rate in recent years was significantly higher than earlier cases (tonsil SCC: 64.7% (55/85) in 2016–2020 vs. 40.4% (19/47) in 2010–2015, *p* = 0.007). Patients with HPV-positive tonsil cancers were significantly younger than those negative (mean [SD]: 58.9 [9.9] vs. 64.3 [13.3] years, *p* = 0.006), but no significant difference was observed between genders. A persistent increase in the incidence of oropharyngeal cancer over the last few decades was observed in Hong Kong, which can be explained by the remarkable increase in HPV-positive tonsil cancers.

## 1. Introduction

The etiological association between infection with high-risk types of human papillomaviruses (HPV) and cancers of the anogenital area including uterine cervix, anus, penis, vagina, and vulva is well known; and there is growing concern over oropharyngeal cancer [1,2,3]. Oropharyngeal cancer is a subtype of head and neck cancer that affects the base of the tongue, palatine tonsils, lingual tonsils, soft palate, uvula, and posterior pharyngeal wall. The accumulated evidence indicates that there are two causal mechanisms for oropharyngeal cancer, one in the common risk factors for other head and neck cancers such as tobacco, alcohol, and pollutants; and the other related to infection with high-risk types of HPV. HPV is sexually acquired, and an early sexual debut, higher number of sexual partners, including oral sex partners, and previous genital warts are associated with an increased risk for HPV-positive oropharyngeal cancer [4,5]. An alert of a cancer epidemic due to HPV-associated oropharyngeal squamous cell carcinoma (OPSCC) especially for the young age group was raised in 2010 [6]. Worldwide, 98,400 new cases and 48,100 deaths of oropharyngeal cancer were estimated in 2020, with age-standardized rates of 1.1 and 0.51 per 100,000 for incidence and mortality, respectively [1]. Reports published over the last decade confirm the increasing burden of OPSCC with high incidence rates observed particularly in Europe, North America, Australia, and New Zealand [1,7,8]; whereas most parts of Asia are still at a relatively low incidence. At present, context-specific data for Hong Kong are still scarcely available [9]. To achieve precise public health planning to combat the possible increase in OPSCC, we set the objectives of this study to depict the temporal trends in changes in the incidence and HPV prevalence in OPSCC over the recent decades in Hong Kong, a metropolitan city in East Asia.

## 2. Materials and Methods

A retrospective study was conducted to determine changes in the incidence of OPSCC and the portion infected with HPV in Hong Kong.

The first part of the study was based on the territory-wide cancer statistics captured by the Hong Kong Cancer Registry [10]. The Hong Kong Cancer registry, established in 1963, is a population-based registry committed to collecting and conducting analyses on data from all cancer cases in Hong Kong, and providing data resources to support the planning and evaluation of cancer services in the healthcare system. The registered cases are primarily based upon local residents’ cases occurring and being diagnosed in the territory, including all types of invasive cancers. The registry collects information on the patient demographics, anatomical site, histology, and stage of every cancer case diagnosed in Hong Kong.

Age-standardized incidence rates of major types of head and neck squamous cell carcinoma (HNSCC) in Hong Kong from 1986 to 2020 obtained from the Hong Kong Registry were analyzed. The age-standardized rate (ASR, per 100,000 population) in accordance with the direct method was calculated by summing up the products of the age-specific rates (*a*_i_, where i denotes the ith age class) and the number of persons (or weight) (*w*_i_) in the same age subgroup i of the chosen reference standard population, then dividing by the sum of the standard population weights [11].

To elucidate any differences between HPV- and non-HPV-associated head and neck cancers, the annual number of new cases of OPSCC and laryngeal SCC were further analyzed. Laryngeal SCC was selected as it shares common risk factors, such as tobacco, with OPSCC; but it is not HPV-associated.

The second part of the study was to examine changes in the proportion of OPSCC that were positive for high-risk HPV DNA. To achieve this, we identified histology-confirmed OPSCC cases that were diagnosed between 2010 and 2020 from four major hospitals. Paraffin-embedded tissues were retrieved for testing. The study was approved by the Joint Chinese University of Hong Kong—New Territories East Cluster Ethics Committee (CREC Ref. No.: 2020.481), and the ethics committees of the hospitals.

The first and last sections of the formalin-fixed paraffin-embedded tumor tissue blocks were examined to ensure a sufficient tumor mass was obtained. For each sample, 5 sections of 5 µm tissue roll were sectioned for DNA extraction using the QIAamp DNA FFPE Tissue Kit (Qiagen, Valencia, CA, USA) following the manufacturer’s protocol.

The quality of extracted DNA was assessed by a real-time PCR targeting the prostaglandin transporter (PGT) gene. A TaqMan primer/probe set targeting the host prostaglandin transporter (PGT) gene 1 was used to monitor the quality of DNA extracted from the FFPE samples. The one-step real-time RT-PCR contained 2 μL of the extracted DNA, 10 μL of 2X TaqMan™ Master Mix (Applied Biosystems, Foster City, CA, USA), 0.15 µM forward primer (5′-ATC CCC AAA GCA CCT GGT TT-3′), 0.15 µM reverser primer (5′-AGA GGC CAA GAT AGT CCT GGT AA-3′), and 0.25 µM probe (5′-FAM-CC ATC CAT G -ZEN- T CCT CAT CTC -IABkFQ-3′) in a final reaction volume of 20 μL. The cycling conditions were 50 °C for 2 min, 95 °C for 10 min, followed by 40 cycles of 95 °C for 15 s and 60 °C for 1 min, and performed with the StepOnePlus Real-Time PCR System (Applied Biosystems, Foster City, CA, USA). Samples were considered negative if the Ct values exceeded 39.9 cycles. Extracted preparations with sufficient DNA quality were submitted for next-generation sequencing to detect HPV DNA.

HPV detection and genotyping was performed using two PCR-based amplicon sequencing assays targeting the conserved L1 open reading frame (ORF) of HPV as previously described [12]. In brief, a pair of dual 12 bp barcodes was indexed to the PCR amplicon using forward and reverse primers for demultiplexing. Short reads generated by Illumina MiSeq PE150 were blasted against a comprehensive PV reference database using UPARSE [13]. An operational taxonomic unit (OTU) count table was created using a 90% identity threshold, assigning each OTU with a PV type. Based on the International Agency for Research on Cancer (IARC) classification, 12 HPV types (HPV16, 18, 31, 33, 35, 39, 45, 51, 52, 56, 58, 59) were ranked as high risk (HR) and considered oncogenic [14].

Confidence intervals of the observed HPV positive rates were calculated using the Wilson score method without continuity correction [15]. Differences in proportion were assessed by Chi-squared test or Fisher’s exact test as appropriate, and the trend of change in positive rates was assessed by the Chi-squared test for trend (Statcalc, Epi Info Version 7.2.5.0, Centre for Disease Control and Prevention, US). Two-tailed P-values less than 0.05 were regarded as statistically significant. Age distribution was assessed by a *t*-test [16].

## 3. Results

**Figure 1:**
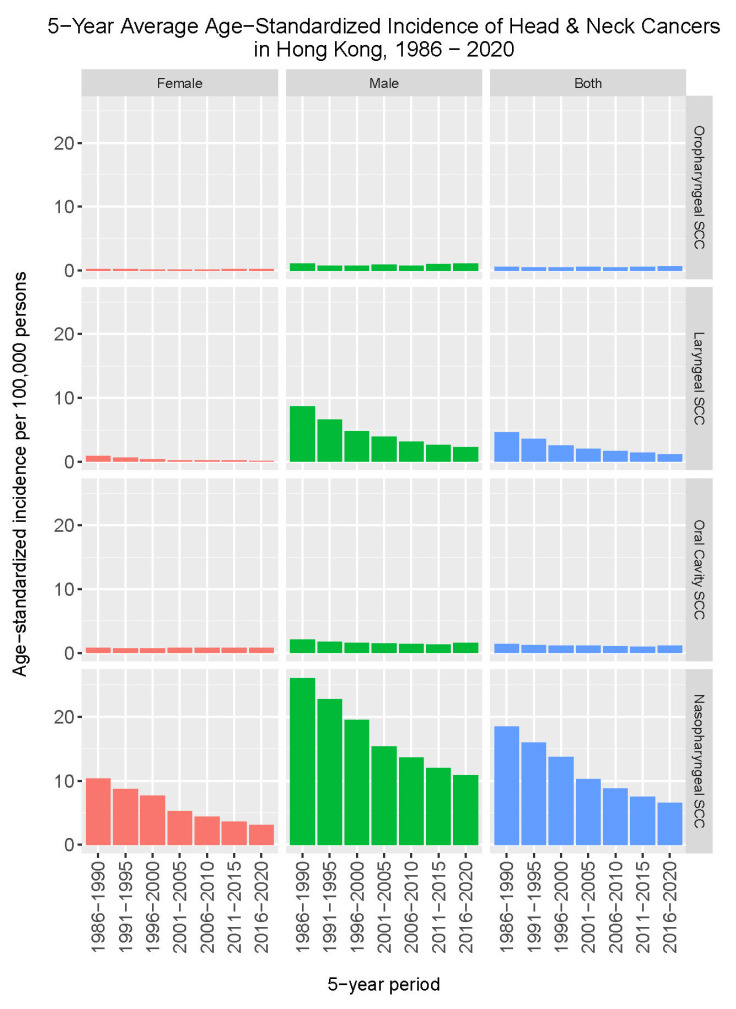
Changes in the incidence of head and neck squamous cell carcinoma (SCC) in Hong Kong, 1986–2020.

Figure 1 shows changes in the incidence rates of major types of head and neck squamous cell carcinoma from 1986 to 2020 in Hong Kong. In contrast to a marked decrease in the incidence rates of nasopharyngeal cancer and laryngeal cancer, where the 5-year average annual age-standardized incidence rates dropped from 18.5 to 6.6 per 100,000 and 4.6 to 1.2 per 100,000, respectively, such a trend of decrease was not observed for cancers of the oropharynx and oral cavity.

**Figure 2:**
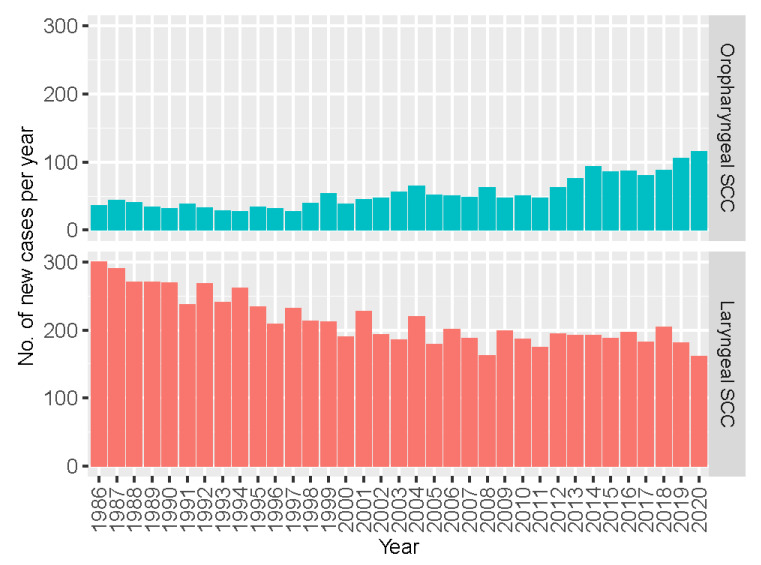
Changes in the number of new cases of oropharyngeal and laryngeal squamous cell carcinoma (SCC) in Hong Kong, 1986–2020.

We then took a closer look at OPSCC with reference to laryngeal SCC that serves as a proxy for non-HPV-associated head and neck cancers. As shown in Figure 2, a contrasting trend of change between these two types of head and neck cancers was noted. The annual number of new cases of OPSCC increased consistently from 36 cases in 1986 to 116 cases in 2020, whereas those of laryngeal cancer decreased from 300 cases to 162 cases.

**Figure 3:**
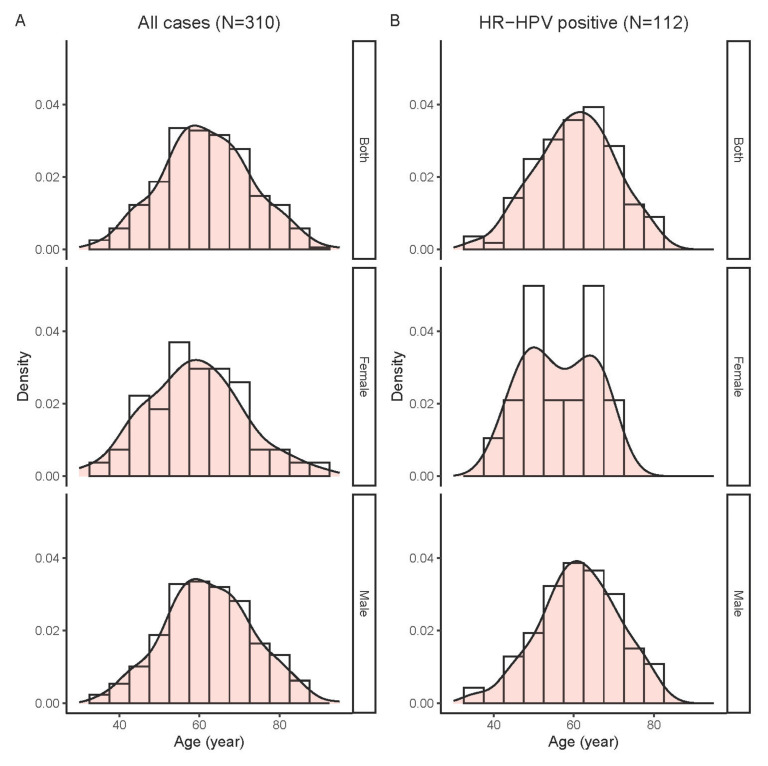
Age distribution of oropharyngeal squamous cell carcinoma (OPSCC) cases tested for human papillomavirus. (**A**) All cases. (**B**) High-risk HPV positive cases.

Since infection with high-risk HPV is a known etiological factor for OPSCC, we further examined changes in the proportion of OPSCC harboring HPV over the past years. In this regard, 310 cases diagnosed between 2010 and 2020 and with adequate DNA quality were analyzed (Table 1). These cases included 132 tonsil SCC and 178 non-tonsil OPSCC from patients aged 31–94 years (mean, 62; standard deviation [SD], 11.4); and the majority were men (256/310, 82.6%). The age distribution of study cases is shown in Figure 3A.

**Figure 4:**
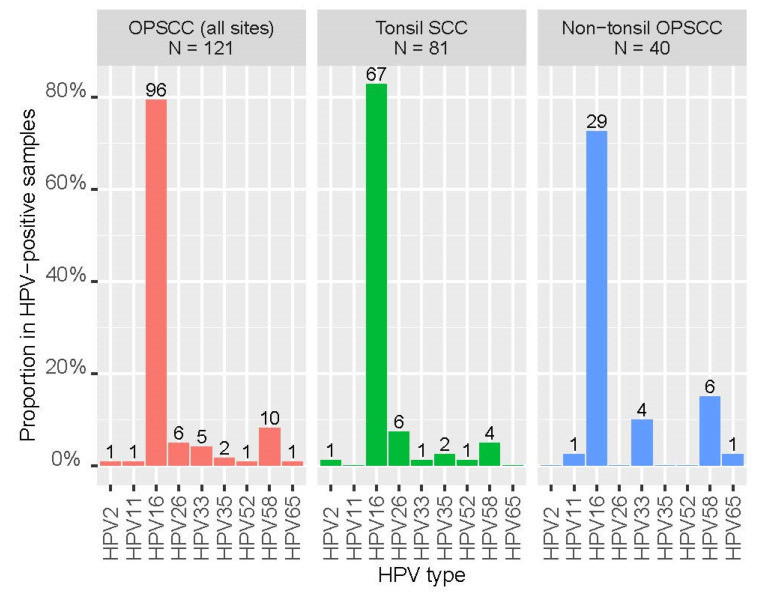
Distribution of human papillomavirus (HPV) types in oropharyngeal squamous cell carcinoma (OPSCC) in Hong Kong.

Of the 310 tumor samples, 121 (39.0%) were positive for HPV DNA with a vast majority (92.6%) 112/121) being high-risk types. The positive rates for high-risk HPV were 36.1%, 56.1%, and 21.3% for all OPSCC, tonsil SCC, and non-tonsil OPSCC, respectively. Two samples harbored co-infection with both HPV16 and HPV58, others were single-type infections. HPV16 was the most common high-risk type, being detected in 79.3% (96/121) of positive samples, followed by HPV58 (10/121, 8.3%), HPV33 (5/121, 4.1%), HPV35 (2/121, 1.7%), and HPV52 (1/121, 0.8%). Of note, HPV26, a type with uncertain carcinogenicity, was detected in six cases of tonsil SCC. The distribution of HPV types in tonsil and non-tonsil SCC is shown in Figure 4.

Two samples with HPV16 and 58 co-infection were counted twice. Non-tonsil OPSCC includes the oropharyngeal wall, tongue base, and soft palate. Numbers above the bars indicate the number of HPV-positive samples.

Further analyses on the correlation between high-risk HPV infection and sex and age were performed. The positive rates for high-risk HPV of all OPSCC cases combined were similar between the two genders (36.3% [93/256] for males, 35.2% [19/54] for females, *p* = 0.874 by Chi-squared test). A comparison based on the male-to-female ratio also did not reveal any significant difference (male-to-female ratio = 4.9 [93/19] for the HPV-positive group; 4.6 [163/35] for the HPV-negative group, *p* = 0.873 by Chi-squared test). There was also no significant difference in age between patient groups positive or negative for high-risk HPV (mean, range [SD]: 60, 35–82 [9.8] vs. 62, 31–94 [12.1] years, *p* = 0.118 by *t*-test). Female, but not male, patients positive for high-risk HPV DNA displayed a bi-modal age distribution (Figure 3B).

Further subgroup analyses restricted to the 132 patients with tonsil SCC revealed that the age of the high-risk HPV-positive group was significantly younger than those of high-risk HPV-negative group (mean, range [SD]: 58.9, 35–79 [9.9] vs. 64.3, 36–94 [13.3] years, *p* = 0.006 by *t*-test), whereas no significant difference between genders was observed (male/female: 4.7 [61/13] vs 4.3 [47/11], *p* = 0.836 by Chi-squared test, for high-risk HPV-positive and -negative groups, respectively).

**Figure 5:**
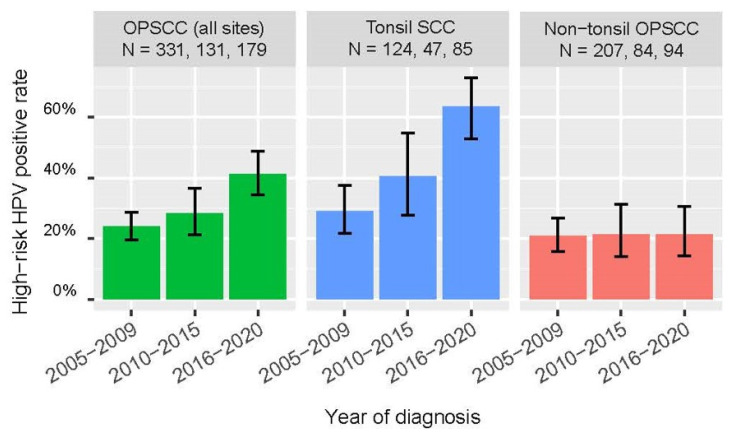
Proportion of human papillomavirus (HPV)-positive oropharyngeal squamous cell carcinoma (OPSCC) from 2005 to 2020.

We observed a significantly higher positive rate for high-risk HPV among OPSCC (all sites combined) and tonsil SCC cases diagnosed between 2016 and 2020 compared to those diagnosed between 2010 and 2015 (all-site OPSCC: 41.9% [95% CI: 34.9–49.2] vs 28.2% [21.2–36.5], *p* = 0.013 by Chi-squared test; tonsil SCC: 64.7% [54.1–74.0] vs. 40.4 [27.6–54.7], *p* = 0.007 by Chi-squared test); whereas no significant increase was observed for non-tonsil SCC (Table 2). Figure 5 incorporated the findings of a previous local study based on similar methodologies to exhibit the trend of change in high-risk HPV-positive rates [17]. Of note, there was a significant linear trend of increase in the proportion of OPSCC (all-site combined) and tonsil SCC positive for high-risk HPV from 2005 to 2020 (*p* < 0.001 for both by Chi-squared test for linear trend).

The positive rate of 2005-2009 was based on a previous local study using similar methodologies [17].

## 4. Discussion

There is a good body of evidence to demonstrate the success of the HPV vaccine in preventing cervical cancer. However, similar efficacy data for oropharyngeal cancer are not yet available. The lack of a readily detectable pre-cancerous stage as an efficacy trial end-point and the long lag time between oral infection and cancer development are the key bottlenecks [18]. Nevertheless, current circumstantial evidence suggests that the HPV vaccine may play a role in preventing oropharyngeal and other HPV-associated head and neck cancers. While several health authorities have included the prevention of HPV-associated head and neck cancers as part of the indication for HPV vaccination, a gender-neutral vaccination program is still not widely available. Since the 2019/20 school year, the Hong Kong SAR government have started to provide free HPV vaccination to Primary Five school girls, but not boys. In this context, solid data on the local trend of change in incidence of HPV-associated head and neck cancers can support an informed public health decision as to whether the HPV vaccination program should be expanded to a gender-neutral one.

It has been estimated that about 30% of oropharyngeal cancers across the globe are driven by HPV [19,20]. In the United Sates, the population-level incidence of HPV-positive oropharyngeal cancers increased by 225% from 1988 to 2004 [21]. In Canada, the proportion of tonsillar cancers that were HPV-positive increased from 25% in 1993–1999 to 62% in 2006–2011 [22]. Both the incidence of oropharyngeal cancer and the proportion positive for HPV varies across Europe. Based on data collected between 1990 and 2012, an estimated 41.9% of all OPSCC cases in Europe are driven by HPV, and this is higher in Northern and Central-Eastern European countries than in Southern and Western European countries [23,24]. Reports from Australia also indicate an increase in OPSCC [25], and the HPV-positive proportion increased from 20% to 63% between 1987 and 2010 [26].

While a notable trend of increase in the incidence of HPV-associated oropharyngeal cancer has been reported across North America, Europe, and Australia, the findings from Asia are more varied. The age-standardized incidence of OPSCC in Asia is on the low side of 0.49 and 0.10 per 100,000 population in males and females, respectively [19]. However, a report from Taiwan showed that both HPV-related and -unrelated head and neck cancers increased during 1995–2009, with the fastest increase in tonsil cancer, particularly among men. The incidence of HPV-related head and neck cancers among Taiwanese men increased from 2.24 per 100,000 in 1995 to 6.15 per 100,000 in 2009, which was similar to those of Western countries [27]. A study from Korea based on the Health Insurance Review and Assessment (HIRA) database showed an increase in male oropharyngeal cancer from 2.7 to 3.1 per 100,000 from 2013 to 2016 [28]. While studies from mainland China and Japan have also reported an increase in incidence, the magnitude of change was relatively small. A study from mainland China based on the Chinese Cancer Registry Annual Report covering 2007–2015 observed an increase in male oropharyngeal cancer incidence by 3.1% annually [29]. In Japan, between 1993 and 2015, an annual 5% increase in the incidence of male oropharyngeal cancer was reported [30].

The current study represents the first of its kind in Hong Kong where changes in the incidence rate, as well as the HPV-positive proportion of oropharyngeal cancer, were examined. The 310 cases available for an HPV test in this study accounted for 34.7% of all OPSCC cases recorded by the Hong Kong Cancer Registry during the study period 2010–2020 [10]. Our findings should be representative of the whole Hong Kong population. We found a remarkable increase in the proportion of tonsil cancers that were positive for high-risk HPV, whereas the positive rate among OPSCC of non-tonsil sites remained unchanged over the years. This is in line with the current understanding that tonsils are the most preferred site within the head and neck region for HPV-mediated carcinogenesis [31,32]. The incidence of OPSCC in Hong Kong and its trend of increase in HPV-positive proportion appear to be lower than Western countries [19]. This could be a result of a difference in exposure to oral HPV infection due to cultural differences in sexual, especially oral, sexual practices. When compared to other Chinese populations in Asia, the magnitude of increase in HPV-associated OPSCC in Hong Kong is faster that mainland China, but slower than Taiwan [27,28,29,30].

Oropharyngeal cancer and laryngeal cancer share common risk factors such as tobacco, and yet the former but not the latter has a strong etiological association with HPV [33,34]. Our finding of an opposite direction of change in incidence of these two cancers further support that HPV is the driving force for the consistent increase in the incidence of oropharyngeal cancer over the last few decades in Hong Kong. Given the strong carcinogenicity of HPV, tobacco control alone is unlikely to achieve a satisfactory effect on combating oropharyngeal cancer. While the current number of new cases of oropharyngeal cancer in Hong Kong is relatively low, at around a hundred cases per year, actions should be taken early to prevent a further upsurge in the coming decades. A gender-neutral HPV vaccination program in Hong Kong is worth considering.

Our study revealed a difference in the distribution of HPV types in oropharyngeal cancer compared to those reported in the West. While HPV16 was the most common type found both in our locality and the West [18,31], HPV58 which is rarely detected in the West was ranked the second in our locality. Such a ranking of high-risk HPV types in oropharyngeal cancer in Hong Kong is reminiscent of cervical cancer [35]. It would be worthwhile to investigate whether HPV58 is also more prevalent in oropharyngeal cancer in other cities in East Asia and Latin America where HPV58 also ranks high in cervical cancer [36,37].

HPV26 is an HPV type with uncertain carcinogenicity. For the purpose of analysis in this study, we classified the six tonsil SCC harboring HPV26 into the non-high-risk HPV group. Of note, HPV26 was reported to be the third most common type, following HPV16 and HPV33, detected from 1090 oropharyngeal cancer cases collected from 29 countries in Europe, Africa, Asia, and America [24]. The true oncogenicity of HPV26 in oropharyngeal cancer deserves further investigation, particularly in view of the lack of coverage of current vaccines for this type.

Our study has limitations. Firstly, there was an underestimation in the incidence of oropharyngeal cancer in Hong Kong since those cancers originating from the base, i.e., posterior one-third, of the tongue could be counted under “tongue” rather “oropharyngeal” squamous cell carcinoma in the Hong Kong Cancer Registry. Secondly, we used a sensitive method to detect HPV DNA that may reveal bystander infections that were not the culprit of carcinogenesis. Our study did not include testing for HPV E6/E7 mRNA and p16 to verify the role of the HPV detected. Nevertheless, the trend of increase in HPV positivity observed in this study is likely a reflection of a genuine increase in HPV attribution in oropharyngeal cancer in Hong Kong over the last few decades.

## 5. Conclusions

The burden of HPV-associated oropharyngeal cancer is escalating worldwide, and Hong Kong is not an exception. Despite the recent advances in new devices such the exoscope and the VITOM 3D system to achieve more precise surgery for the head and neck region [38], primary prevention is still an important aspect. While a great effort has been focused on the control of tobacco consumption, the other important carcinogen, HPV infection, should not be neglected. Research studies to generate more direct and indirect evidence on the protection offered by HPV vaccination would enhance an evidence-based decision of policy makers to broaden the scope of vaccination programs against other health needs. With the increase in HPV-associated OPSCC, the development of biomarker panels incorporating HPV DNA/mRNA testing for early cancer screening is an urgent priority [39,40]. Given the difference in epidemiology, available infrastructure, and quality of life issues, management guidelines and public health strategies developed in the West may not be the best for Asian patients [41]. Further studies focused on monitoring the trend of change in HPV-associated OPSCC, the efficacy of de-escalation of treatment, and including HPV genotyping information in cancer registries, particularly of Asian countries and cities, are needed. Our study documented and highlighted the increase in the disease burden of HPV-associated oropharyngeal cancer, which supports the need for a gender-neutral HPV vaccination program in Hong Kong.

## Figures and Tables

**Table 1:** Characteristics of 310 oropharyngeal squamous cell cancer (OPSCC) cases examined for HPV DNA.

Characteristic	Gender Distribution, *N* (%)		
	Total (*N* = 310)	Male (*N* = 256)	Female (*N* = 54)
Age group (years)			
31–40	7 (2.3)	5 (2.0)	2 (3.7)
41–50	41 (13.2)	30 (11.7)	11 (20.4)
51–60	100 (32.3)	83 (32.4)	17 (31.5)
61–70	95 (30.6)	79 (30.9)	16 (29.6)
≥71	67 (21.6)	59 (23.0)	8 (14.8)
Subsite			
Tonsil	132 (42.9)	108 (42.2)	24 (44.4)
Tongue base	111 (35.8)	90 (35.2)	21 (38.9)
Oropharynx	42 (13.5)	37 (14.5)	5 (9.3)
Soft palate	25 (8.1)	21 (8.2)	4 (7.4)

**Table 2:** Proportion of oropharyngeal cancers positive for high-risk human papillomavirus according to the year of diagnosis.

	Oropharyngeal Squamous Cell Carcinoma (OPSCC), No. Positive for High-Risk HPV/No. Tested (%)		
Year of Diagnosis	All Sites within Oropharynx	Tonsil	Non-Tonsil
2010	3/15 (20.0)	1/3 (33.3)	2/12 (16.7)
2011	6/18 (33.3)	3/5 (60.0)	3/13 (23.1)
2012	6/19 (31.6)	3/8 (37.5)	3/11 (27.3)
2013	6/26 (23.1)	4/11 (36.4)	2/15 (13.3)
2014	6/21 (28.6)	4/11 (36.4)	2/10 (20.0)
2015	10/32 (31.3)	4/9 (44.4)	6/23 (26.1)
2010–2015	37/131 (28.2)	19/47 (40.4)	18/84 (21.4)
2016	8/30 (26.7)	6/12 (50.0)	2/18 (11.1)
2017	19/35 (54.3)	15/17 (88.2)	4/18 (22.2)
2018	9/32 (28.1)	6/14 (42.9)	3/18 (16.7)
2019	17/40 (42.5)	11/18 (61.1)	6/22 (27.3)
2020	22/42 (52.4)	17/24 (70.8)	5/18 (27.8)
2016–2020	75/179 (41.9)	55/85 (64.7)	20/94 (21.3)

## Data Availability

The cancer statistics data underlying this study are available in the public domain: https://www3.ha.org.hk/cancereg/default.asp (accessed on 1 September 2023).
